# Relationship between NaCl- and H_2_O_2_-Induced Cytosolic Ca^2+^ Increases in Response to Stress in *Arabidopsis*

**DOI:** 10.1371/journal.pone.0076130

**Published:** 2013-10-04

**Authors:** Zhonghao Jiang, Shan Zhu, Rui Ye, Yan Xue, Amelia Chen, Lizhe An, Zhen-Ming Pei

**Affiliations:** 1 The Key Laboratory of Cell Activities and Stress Adaptations, School of Life Science, Lanzhou University, Lanzhou, Gansu, China; 2 Center on Plant Environmental Sensing, Institute for Global Change, College of Life and Environmental Sciences, Hangzhou Normal University, Hangzhou, Zhejiang, China; 3 Department of Biology, Duke University, Durham, North Carolina, United States of America; Wake Forest University, United States of America

## Abstract

Salinity is among the environmental factors that affect plant growth and development and constrain agricultural productivity. Salinity stress triggers increases in cytosolic free Ca^2+^ concentration ([Ca^2+^]_i_) via Ca^2+^ influx across the plasma membrane. Salinity stress, as well as other stresses, induces the production of reactive oxygen species (ROS). It is well established that ROS also triggers increases in [Ca^2+^]_i_. However, the relationship and interaction between salinity stress-induced [Ca^2+^]_i_ increases and ROS-induced [Ca^2+^]_i_ increases remain poorly understood. Using an aequorin-based Ca^2+^ imaging assay we have analyzed [Ca^2+^]_i_ changes in response to NaCl and H_2_O_2_ treatments in *Arabidopsis thaliana*. We found that NaCl and H_2_O_2_ together induced larger increases in [Ca^2+^]_i_ in *Arabidopsis* seedlings than either NaCl or H_2_O_2_ alone, suggesting an additive effect on [Ca^2+^]_i_ increases. Following a pre-treatment with either NaCl or H_2_O_2_, the subsequent elevation of [Ca^2+^]_i_ in response to a second treatment with either NaCl or H_2_O_2_ was significantly reduced. Furthermore, the NaCl pre-treatment suppressed the elevation of [Ca^2+^]_i_ seen with a second NaCl treatment more than that seen with a second treatment of H_2_O_2_. A similar response was seen when the initial treatment was with H_2_O_2_; subsequent addition of H_2_O_2_ led to less of an increase in [Ca^2+^]_i_ than did addition of NaCl. These results imply that NaCl-gated Ca^2+^ channels and H_2_O_2_-gated Ca^2+^ channels may differ, and also suggest that NaCl- and H_2_O_2_-evoked [Ca^2+^]_i_ may reduce the potency of both NaCl and H_2_O_2_ in triggering [Ca^2+^]_i_ increases, highlighting a feedback mechanism. Alternatively, NaCl and H_2_O_2_ may activate the same Ca^2+^ permeable channel, which is expressed in different types of cells and/or activated via different signaling pathways.

## Introduction

The presence of high salinity affects almost every aspect of plant growth and development, and causes enormous losses in agricultural production worldwide. It is estimated that about 10 million hectares of agricultural land is abandoned every year because of high salinity, and salt stress affects as much as a quarter to a third of global agricultural land, particularly land which has been irrigated [1-3]. Given the continued increase in human population occurring in the world, it is estimated that crop production must be increased 50% by 2025 to stave off large-scale food shortages [4]. Thus, it is crucial to understand how plants respond to salt stress.

Many studies have been carried out to dissect the molecular and genetic mechanisms of the plant response to salt (NaCl) stress, often using the model organism *Arabidopsis thaliana* [5-7]. Excess NaCl is toxic to plants, causing cellular ion imbalances and hyperosmotic stress [1-3,7]. NaCl stress also triggers a calcium signaling cascade in plants, leading to transcriptional regulation and subsequent physiological and developmental responses [1]. Although the molecular nature of initial perception of salt stress is unknown, it has been well established that salt stress triggers a transient increase in cytosolic Ca^2+^ concentration ([Ca^2+^]_i_) that lasts about 2 min [8,9]. This increase has been proposed to represent a salt sensory process in plants [3,10].

In plants, Ca^2+^ as a secondary messenger is a key element to understanding a sophisticated network of signaling pathways responding to a large array of abiotic and biotic stimuli, including salt stress [11-13]. These speciﬁc Ca^2+^ signatures are formed by the tightly regulated activities of Ca^2+^ channels and transporters in different tissues, organelles and membranes [13-16], and the changes in [Ca^2+^]_i_ are detected by cytosolic Ca^2+^ sensors. More than 250 Ca^2+^-binding EF-hand proteins have been identified in *Arabidopsis* [17], including the calmodulin (CaM), the calmodulin-like (CML), the Ca^2+^-dependent protein kinase (CDPK), and the calcineurin B-like (CBL) protein families. These cytosolic Ca^2+^ sensors decode and relay the information encoded within [Ca^2+^]_i_ signatures, allowing the plant to tightly bring about the appropriate adaptation to its ever-changing environment.

The salinity stress-induced increase in [Ca^2+^]_i_ leads to the activation of SOS3/CBL4, which functions as the primary Ca^2+^ sensor of [Ca^2+^]_i_ changes under salt stress [3]. Upon activation, SOS3/CBL4 interacts with the C-terminal region of a CBL-interacting protein kinase (CIPK) called SOS2/CIPK24, which in turn activates a plasma membrane Na^+^/H^+^ antiporter SOS1 that transports sodium ions out of the cell [3]. This salt signaling pathway reinforces the concept that the salt-induced [Ca^2+^]_i_ increase is an essential component for bringing about the plant response to salt tress.

Interestingly, after salt stress treatment there is an overproduction of reactive oxygen species (ROS), such as hydrogen peroxide (H_2_O_2_) [18-22]. The time constants for salt-induced increases in [Ca^2+^]_i_ and ROS are about 3 sec and 400 sec, respectively, as estimated from previous studies [8,21]. It appears that the increase in [Ca^2+^]_i_ occurs earlier than the ROS elevation after salt stress treatment. Considering ROS have also been shown to trigger increases in [Ca^2+^]_i_ [21,23-26], it is possible that ROS-induced [Ca^2+^]_i_ increases might serve as a feed forward mechanism in the salt stress signal transduction pathway. However, less is known about the relationship and interaction between the salt stress-induced [Ca^2+^]_i_ increases and the [Ca^2+^]_i_ increases evoked by ROS, which are produced in response to either salt stress specifically or other stresses in general [1,27].

In this study, we have systematically analyzed the relationship and interaction between salt stress-induced [Ca^2+^]_i_ increases and the ROS-induced [Ca^2+^]_i_ increases in *Arabidopsis*. We found the increases in [Ca^2+^]_i_ induced by both stimuli were higher than these induced by either single stress, suggesting that NaCl and H_2_O_2_ have an additive effect on [Ca^2+^]_i_. We have also found that NaCl-induced [Ca^2+^]_i_ increases might inhibit both NaCl- and H_2_O_2_-gated channels by a feedback mechanism, but more NaCl-gated channels; a similar response was seen when the H_2_O_2_-induced [Ca^2+^]_i_ increases were analyzed. These data suggest responses seen involve both feedback inhibitory mechanisms, as well as an interaction between two stimuli-mediated Ca^2+^ signaling pathways.

## Results

### Dose-dependence and kinetics of NaCl- and H_2_O_2_-induced [Ca^2+^]_i_ increases

**Figure 1:**
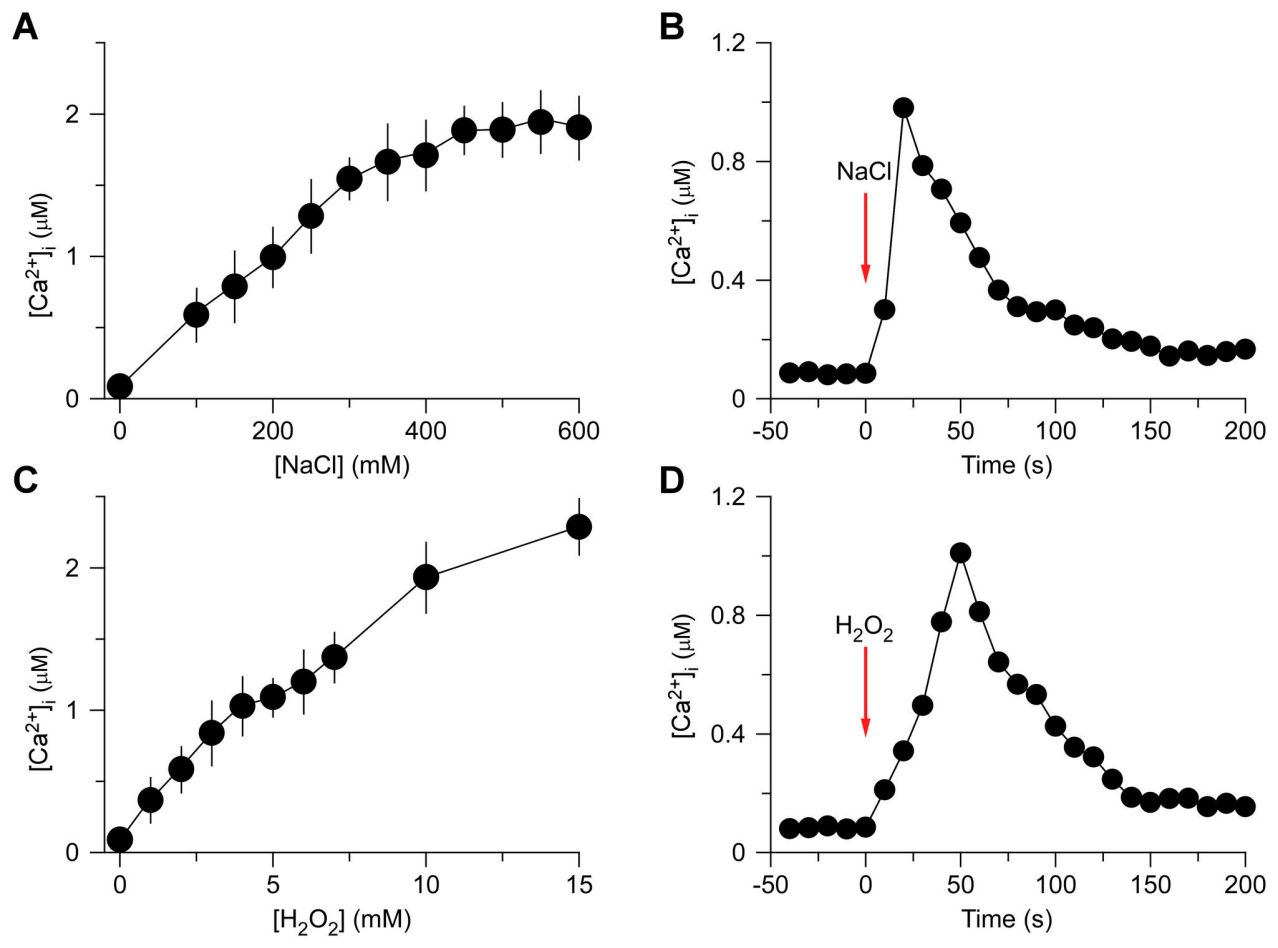
Increases in [Ca^2+^]_i_ in response to NaCl and H_2_O_2_ treatments. (A and C) Increases in [Ca^2+^]_i_ induced by several concentrations of NaCl (A) and H_2_O_2_ (C) in *Arabidopsis*. Seedlings expressing aequorin and grown for 7 days were treated with solutions containing several concentrations of NaCl or H_2_O_2_, and aequorin images were taken every 10 sec for 500 sec. Data for four independent experiments are shown (mean ± sem; *n* = 64). (B and D) Time courses of increases in [Ca^2+^]_i_ induced by 200 mM NaCl (B) or 4 mM H_2_O_2_ (D). Seedlings grown for 7 days were treated with NaCl and H_2_O_2_ at time zero, and aequorin images were taken every 10 sec. Representative recordings from individual seedlings were shown. Similar results were seen in six independent experiments using 128 seedlings.

To analyze whether and how increases in [Ca^2+^]_i_ induced by NaCl and H_2_O_2_ interact each other in *Arabidopsis*, we attempted initially to identify optimum concentrations of NaCl and H_2_O_2_, which ideally could be applied to generate about half of the maximum amplitude of [Ca^2+^]_i_ for potential up- and down-regulation. In addition, we attempted to establish the kinetics of NaCl- and H_2_O_2_-induced increases in [Ca^2+^]_i_ for administrating these stresses in different sequential combinations. First, to analyze NaCl-induced increases in [Ca^2+^]_i_, we treated *Arabidopsis* seedlings expressing aequorin with solutions containing 0 to 600 mM NaCl. Aequorin bioluminescence images were taken every 10 sec for 500 sec, and the peak [Ca^2+^]_i_ was calculated and analyzed, as NaCl induces a transient increase in [Ca^2+^]_i_ [8,9]. Plants grown on the half-strength MS medium had an average basal [Ca^2+^]_i_ of 80 ± 21 nM (Figure 1A and C). As expected, the [Ca^2+^]_i_ increased in response to NaCl treatment (Figure 1A). The magnitudes of [Ca^2+^]_i_ increases were dependent on the concentration of NaCl, higher concentration of NaCl evoked a larger increase in [Ca^2+^]_i_. The NaCl concentration needed for a half-maximal response was ^~^200 mM, which was chosen as an optimum concentration to subsequently analyze the interaction with H_2_O_2_-induced increases in [Ca^2+^]_i_.

Then, we determined the temporal dynamics of NaCl-induced [Ca^2+^]_i_ increases under the imposed experimental conditions as a control for further comparison (Figure 1B). We found that [Ca^2+^]_i_ increased immediately after the application of 200 mM NaCl, reached a peak of ^~^1 µM at about 20 sec, and then declined gradually (Figure 1B). Note that, the peaking time might be shorter than 20 sec based on previous studies [8,9]. Nevertheless, imaging aequorin bioluminescence for less than 10 sec resulted in images with low a signal-noise ratio in our system. Thus, the temporal resolution was about 10 sec, which is sufficient for the current study. At about 200 sec, the [Ca^2+^]_i_ was reduced to a new resting level of under 200 nM.

Similarly, we analyzed increases in [Ca^2+^]_i_ in response to H_2_O_2_. Seedlings were treated with different concentrations of H_2_O_2_ from 0 to 15 mM and [Ca^2+^]_i_ was analyzed. As expected, H_2_O_2_ induced increases in [Ca^2+^]_i_ in a dose-dependent manner (Figure 1C). The H_2_O_2_ concentration for a half-maximal response was around 4 mM with the magnitude of [Ca^2+^]_i_ similar to that induced by 200 mM NaCl. We then determined the temporal dynamics of the [Ca^2+^]_i_ increase induced by 4 mM H_2_O_2_. Following treatment with 4 mM H_2_O_2_, [Ca^2+^]_i_ increased and reached a peak of ^~^1 µM at 50 sec (Figure 1D). It took another 100 sec for the [Ca^2+^]_i_ to reach a new basal level of just under 200 nM. Taken together, it seems that increases in [Ca^2+^]_i_ occur faster in response to NaCl than H_2_O_2_, but are reset to a resting level 150 sec after treatment.

### Additive effect of NaCl and H_2_O_2_ on triggering increases in [Ca^2+^]_i_

**Figure 2:**
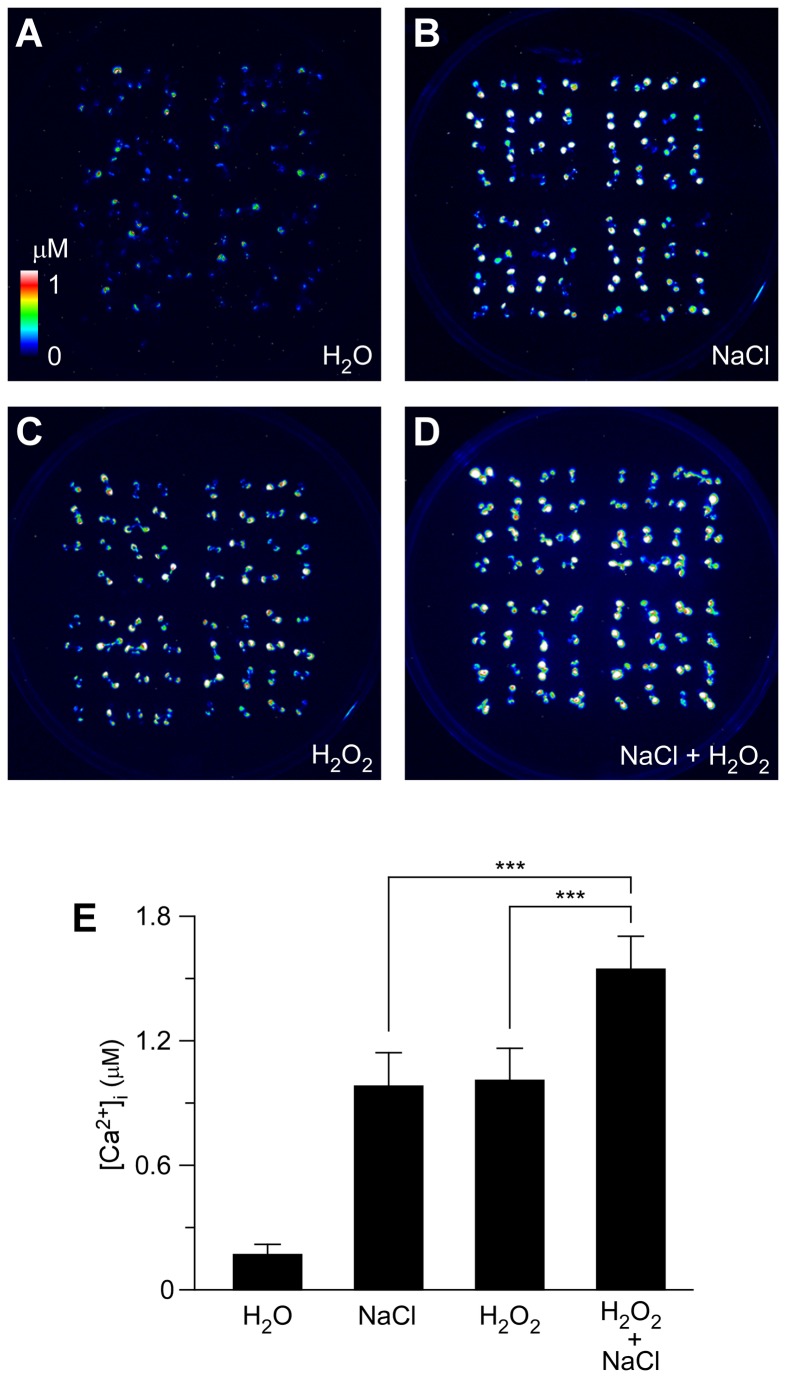
Increases in [Ca^2+^]_i_ in response to NaCl and H_2_O_2_ individually or combined. (A to D) Imaging of [Ca^2+^]_i_ increases in response to the treatments of water (H_2_O; A), 200 mM NaCl (B), 4 mM H_2_O_2_ (A), and 200 mM NaCl and 4 mM H_2_O_2_ together (D) in *Arabidopsis* seedlings expressing aequorin. [Ca^2+^]_i_ increases were analyzed by imaging bioluminescence and scaled by a pseudo-color bar. (E) Quantification of [Ca^2+^]_i_ increases from experiments as in (A) to (D). Data for four independent experiments are shown (mean ± sd; *n* = 64; *** *P* < 0.001; NS, not significant *P* > 0.05).

To investigate thoroughly the relationship and/or interaction between [Ca^2+^]_i_ increases triggered by NaCl and H_2_O_2_, *Arabidopsis* seedlings were treated with 200 mM NaCl or 4 mM H_2_O_2_ separately, or 200 mM NaCl together with 4 mM H_2_O_2_. Note that, although salt-induced ROS production could be detected within ^~^2 min after salt treatment [21] the estimated half-time to the peak of ROS production is more than 5 min. Note also that, we did not detect a second peak of [Ca^2+^]_i_ within 5 min after salt stress treatment (Figure S1A), suggesting that salt-induced ROS could not trigger a detectable increase in [Ca^2+^]_i_ under the current experimental conditions. Because we measured [Ca^2+^]_i_ changes within 5 min after salt treatment, the effect of salt-induced ROS on [Ca^2+^]_i_ should not interfere apparently. The [Ca^2+^]_i_ increases recorded after single treatments were consistent with the results described above (Figure 2A–C). NaCl and H_2_O_2_ induced similar increases in [Ca^2+^]_i_ (Figure 2E). When plants were treated with both stimuli simultaneously, the peaks of [Ca^2+^]_i_ were much larger than that induced by each individual stimulus (Figure 2D and E), showing an additive effect. To further analyze how salt-induced ROS affects [Ca^2+^]_i_ increases in response to salt stress within 500 sec, we carried out an experiment by using the NADPH oxidase inhibitor DPI [28] and ROS scavengers ascorbic acid and glutathione [27], and found that neither of these reagents significantly affected [Ca^2+^]_i_ increases induced by NaCl (Figure S1). These results suggest that the NaCl- and H_2_O_2_-induced [Ca^2+^]_i_ increases may be largely independent events. In other words, NaCl and H_2_O_2_ might activate different Ca^2+^ permeable channels.

### The crosstalk between NaCl- and H_2_O_2_-induced [Ca^2+^]_i_ increases

**Figure 3:**
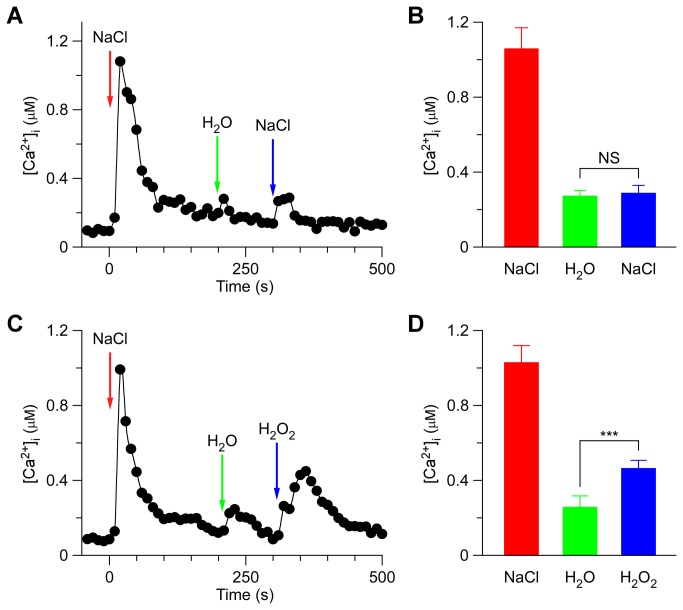
NaCl-induced [Ca^2+^]_i_ increases inhibits NaCl-activated channels more than H_2_O_2_-activated channels. (A and C) *Arabidopsis* seedlings were subjected to a 200 mM NaCl treatment once at 0 sec, and the solution was perfused by deionized water at 200 sec. Then, a second 200 mM NaCl (A), or 4 mM H_2_O_2_ (C) treatment was applied around 300 sec. Aequorin luminescence was recorded continuously through the treatments in the dark. (B and D) Quantification of [Ca^2+^]_i_ increases for the 2^nd^ NaCl (B) or 2^nd^ H_2_O_2_ treatment (C) from experiments as in (A) to (C), respectively. Data for four independent experiments are shown (mean ± sd; *n* = 64; NS, not significant *P* > 0.05; *** *P* < 0.001).

To further characterize the potential interaction between the two stimuli-triggered [Ca^2+^]_i_ signals, plants were successively treated either with the same stimulus or the other. When the *Arabidopsis* seedlings were treated with 200 mM NaCl, the level of [Ca^2+^]_i_ increased quickly to reach a peak and decreased to the new resting level after 150 sec (Figure 3A), as described in Figure 1B. A subtle increase in [Ca^2+^]_i_ could be detected in seedlings after washing with deionized water at 200 sec (Figure 3A and B; green). Then, NaCl was added again, which caused a small increase in [Ca^2+^]_i_. It decayed from 300 sec to a level similar to the previous resting level (Figure 3A and B). Compared to the first NaCl treatment, which led to a large [Ca^2+^]_i_ increase to ^~^1 µM, the 2^nd^ NaCl treatment resulted in a [Ca^2+^]_i_ increase that was only a fraction of the size of the first [Ca^2+^]_i_ increase. This observation suggests that the NaCl-activated Ca^2+^ permeable channel (NaC) might be desensitized or adapted by unknown signaling elements upstream of NaC activation. To test whether the NaC is desensitized or adapted, we waited for 3 hr and were able to detect a normal (^~^1 µM) [Ca^2+^]_i_ increase in response to NaCl, suggesting that the NaC is most likely desensitized (data not shown).

Subsequently we analyzed whether the hydrogen peroxide-activated Ca^2+^ permeable channel (HpC) was affected by the initial NaCl treatment. The second NaCl treatment was replaced by 4 mM H_2_O_2_ at 300 sec (Figure 3C). Interestingly, the peak of [Ca^2+^]_i_ induced by H_2_O_2_ was clearly greater than that induced by 200 mM NaCl (*P* < 0.001). After 450 sec, the [Ca^2+^]_i_ decreased to a new basal level under 200 µM (Figure 3C and D). The lower inhibition of HpC than NaC by the initial NaCl treatment suggests that the initial high level of [Ca^2+^]_i_, which resulted from NaC activation (called ^NaC^[Ca^2+^]_i_ microdomain/puff) subsequently inhibited NaC more than HpC (Figure 3).

**Figure 4:**
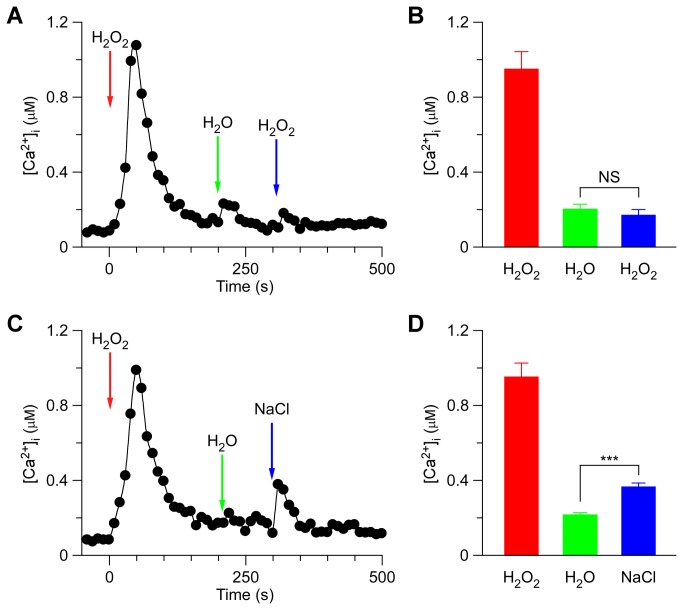
H_2_O_2_-induced [Ca^2+^]_i_ increases inhibits H_2_O_2_-activated channels more than NaCl-activated channels. (A and C) *Arabidopsis* seedlings were subjected to a 4 mM H_2_O_2_ treatment once at 0 sec, and the solution was perfused by deionized water at 200 sec. Then, a second 4 mM H_2_O_2_ (A), or 200 mM NaCl (C) treatment was applied around 300 sec. Aequorin luminescence was recorded continuously through the treatments in the dark. (B and D) Quantification of [Ca^2+^]_i_ increases for the 2^nd^ H_2_O_2_ (B) or 2^nd^ NaCl treatment (C) from experiments as in (A) to (C), respectively. Data for four independent experiments are shown (mean ± sd; *n* = 64; NS, not significant *P* > 0.05; *** *P* < 0.001).

**Figure 5:**
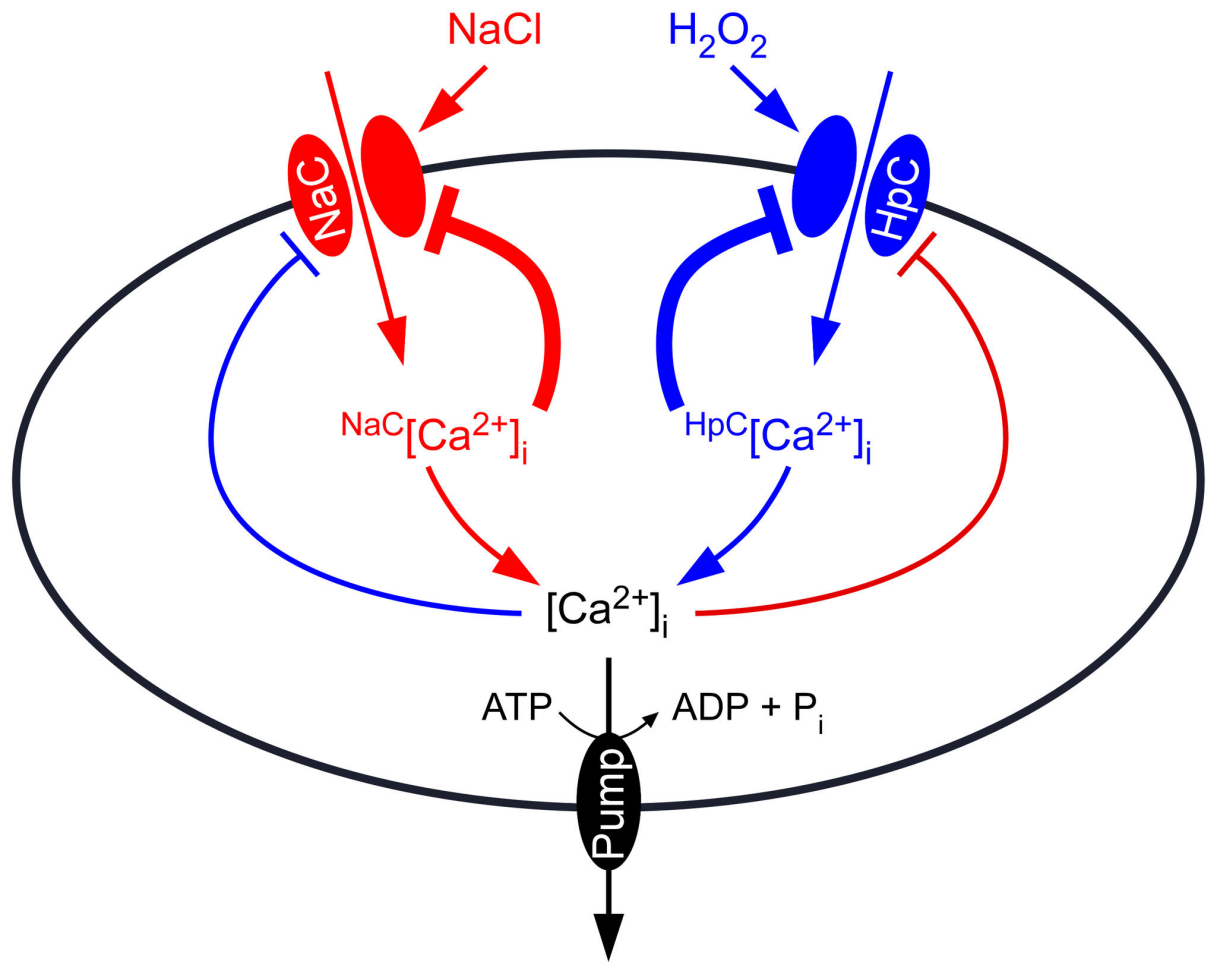
Model for the interaction between NaCl- and H_2_O_2_-induced [Ca^2+^]_i_ increases. A Ca^2+^ channel activated by NaCl (NaC) results in localized [Ca^2+^]_i_ increases, called NaC-related [Ca^2+^]_i_ microdomain (^NaC^[Ca^2+^]_i_). ^NaC^[Ca^2+^]_i_ feedback inhibits the activity of NaC. HpC, a Ca^2+^ channel activated by hydrogen peroxide, leads to localized [Ca^2+^]_i_ increases, called ^HpC^[Ca^2+^]_i_ microdomain. ^HpC^[Ca^2+^]_i_ also feedback inhibits HpC activity. The [Ca^2+^]_i_ mircodomain-mediated inhibition of Ca^2+^ channels is the major feedback inhibitory pathways (thick lines). In addition, both ^NaC^[Ca^2+^]_i_ and ^HpC^[Ca^2+^]_i_ might contribute to a global [Ca^2+^]_i_ increase, which further inhibits both NaC and HpC, serving as global feedback inhibitory pathways (thin lines). [Ca^2+^]_i_ is reset to the resting level by plasma membrane Ca^2+^ pumps.

By analogy, we used H_2_O_2_ as the first stimuli to treat the seedlings and then analyzed the second treatment using H_2_O_2_ or NaCl (Figure 4). When H_2_O_2_ was added to the Petri dish, at 300 sec after the first H_2_O_2_ treatment, the [Ca^2+^]_i_ level stabilized at 178 ± 32 nM, similar to previous resting levels (Figure 4A and B). But when we used 200 mM NaCl to replace H_2_O_2_ at 300 sec, the peak values of [Ca^2+^]_i_ were 381 ± 23 nM, small but significantly higher than that seen with the second H_2_O_2_ treatment (Figure 4B and D). Similarly, our results suggest that the high [Ca^2+^]_i_, which resulted from the initial HpC activation (called ^HpC^[Ca^2+^]_i_ microdomain), inhibited HpC more than NaC (Figures 4 and 5).

## Discussion

Calcium is a universal second messenger that plays an important role in signal transduction in animals and plants [25,29-31]. In the past 20 years, tremendous progress has been made in understanding the changes in [Ca^2+^]_i_ that appear in response to various abiotic and biotic stresses in plants, including salt stress, oxidative stress, drought, high and low temperatures, and pathogen elicitors [13,25,26,32]. It is known that a specific stimulus can trigger unique temporal and spatial patterns of [Ca^2+^]_i_, also known as [Ca^2+^]_i_ signatures [33]. The [Ca^2+^]_i_ signature encodes information from the environmental stimulus which will be decoded subsequently by intracellular Ca^2+^ sensors, such as calmodulins (CaMs) and calcineurin B-like proteins (CBLs), leading to the activation of downstream events [10]. It is also known that the basal [Ca^2+^]_i_ is maintained at a concentration about 10,000-fold below the extracellular Ca^2+^ concentration [29,31,34]. In general, Ca^2+^ channels in the plasma membrane and/or endomembranes are activated in response to environmental stimuli, leading to increases in [Ca^2+^]_i_ [30,32]. Salt stress-induced increases in [Ca^2+^]_i_ have long been proposed as being involved in the process perceiving the salt signal although the properties of the salt-activated Ca^2+^ permeable channel are poorly understood and its molecular nature remains to be identified [1,2,7].

In addition, various abiotic and biotic stresses lead to the production of ROS and oxidative stresses, which control many different processes in plants [27,35-37]. It has been well established that salt stress enhances the production of reactive oxygen species (ROS) in plants [18-22]. Interestingly, ROS has also been shown to activate Ca^2+^ permeable channels in the plasma membrane, which in turn lead to Ca^2+^ influx into the cell and thus increases in [Ca^2+^]_i_ [19,24,38]. Note that, the salt stress-induced [Ca^2+^]_i_ increases precede the production of H_2_O_2_ signaling molecule [39]. Nevertheless, little is known about the molecular mechanisms underlying ROS perception in plant cells, and it is possible that ROS activation of Ca^2+^ permeable channels may serve as a ROS perception process.

The decay of the increases in [Ca^2+^]_i_ induced by both NaCl and H_2_O_2_ seen in this study (Figure 1B and D) as well as previous studies [8,9,15] indicates that the stimulus-activated Ca^2+^ permeable channels may be inactivated via a feedback inhibitory mechanism, i.e. elevated [Ca^2+^]_i_ inhibits these channels, a desensitization process commonly seen for receptor ion channels in animals [31,40]. It remains to be addressed whether the localized increases in [Ca^2+^]_i_ induced by one stimulus, called [Ca^2+^]_i_ microdomain [29,31], inhibit the other stimulus-activated Ca^2+^ permeable channels. It is known that that NaCl induces multiple peaks of [Ca^2+^]_i_ under certain conditions [9], possibly because the same NaCl-sensitive channels are repetitively activated, or that NaCl might trigger H_2_O_2_ production which subsequently activates another Ca^2+^ channel, different from the NaCl-sensitive channel. Under our experimental conditions, we did not observe multiple peaks of [Ca^2+^]_i_ after salt treatment (Figure S1A).

NaCl and H_2_O_2_ together induced larger increases in [Ca^2+^]_i_ than either NaCl or H_2_O_2_ alone (Figure 2), suggesting that NaCl and H_2_O_2_ may activate distinct Ca^2+^ permeable channels, NaC and HpC (Figure 5). NaC and HpC are likely regulated by feedback inhibition (Figure 5), considering their desensitization seen in this study (Figure 1B and D) as well as previous reports [8,9]. We demonstrated that repetitive NaCl treatments failed to trigger repetitive [Ca^2+^]_i_ increases (Figure 3A and B). This indicates that the NaC cannot be activated repetitively within a short period of time, i.e. NaC is possibly desensitized. We propose that a feedback inhibition may be involved in the desensitization (Figure 5). Upon NaCl treatment, the NaC opens, leading to the localized increase of [Ca^2+^]_i_, ^NaC^[Ca^2+^]_i_ microdomain/puff. ^NaC^[Ca^2+^]_i_ in turn signals the channel to close, which prevents further [Ca^2+^]_i_ increases and allows the basal [Ca^2+^]_i_ to be reset via Ca^2+^ pumps. This feedback inhibition avoids the excessive increase of [Ca^2+^]_i_ which could be disastrous to plant cells. The same phenomenon was also observed with the activation of HpC (Figure 5), i.e. ^HpC^[Ca^2+^]_i_ microdomain inhibits HpC via a feedback mechanism. Clearly, the most significant effect is that after the initial treatment by either NaCl or H_2_O_2_, [Ca^2+^]_i_ increases induced by both NaCl and H_2_O_2_ are reduced (Figures 3 and 4). It is most likely that localized ^NaC^[Ca^2+^]_i_ and ^HpC^[Ca^2+^]_i_ merge to form a relatively global [Ca^2+^]_i_, which then feedback inhibits both NaC and HpC (Figure 5). We observed that *Arabidopsis* was unable to recover from 200 mM NaCl treatment 5 min after an initial stimulation. Similar results were observed after 4 mM H_2_O_2_ treatment. In contrast, a previous study has shown that *Arabidopsis* is able to recover its ability to respond almost fully to cold shock 3 min after an initial cold shock [41]. Note that, our work does not prove that HpC and NaC are localized in discreet and different microdomains in the plasma membrane, rather we have shown HpC and NaC may differ and interact via [Ca^2+^]_i_ microdomains. It is also possible that NaCl and H_2_O_2_ may activate the same Ca^2+^ permeable channel, which is expressed in different types of cells and/or activated via different signaling pathways, leading to the differential changes in [Ca^2+^]_i_.

In general, when plants are exposed to one stress, their resistance to other stresses can be enhanced. It is most likely that stress-evoked [Ca^2+^]_i_ increases as well as stress-stimulated overproduction of ROS function as key integrators, possibly mediating stress signal perception and signal transduction. Our results demonstrate the inhibitory interaction of NaCl- and H_2_O_2_-induced [Ca^2+^]_i_ increases, and may predict distinct Ca^2+^ permeable channels activated by NaCl and H_2_O_2_, respectively (Figure 5). In the future, it is important to analyze the pharmacological properties of these putative Ca^2+^ permeable channels activated by NaCl and H_2_O_2_ as described previously for MAMP-activated channels [42]. Obviously, the identification of these channels or sensors will be a hallmark in the study of plant salt resistance in the future. In addition, how NaC and HpC interact to contribute to the [Ca^2+^]_i_ signatures and other downstream events can be further analyzed when their molecular nature is identified.

## Materials and Methods

### Plant materials and growth conditions

*Arabidopsis thaliana* ecotype Columbia-0 (Col-0) constitutively expressing intracellular aequorin (pMAQ2, a kind gift from Dr. M. Knight) under the control of the cauliflower mosaic virus 35S promoter was used [8,33]. *Arabidopsis* plants were grown in 150 mm x 15 mm round Petri dishes in half-strength Murashige and Skoog salts (MS; Gibco), supplemented with 1.5% (w/v) sucrose (Sigma), and 0.8% (w/v) agar (Becton Dickinson) adjusted to pH 6.0 with KOH in controlled environmental rooms at 21 ± 2°C. The fluency rate of white light was ^~^110 µmol m^-2^ sec^-1^. The photoperiods were 16 h light/8 h dark cycles. Seeds were sterilized with 2.5% PPM (Plant preservative mixture; Caisson Laboratories) and stratified at 4°C for 3 days in the dark, and then transferred to growth rooms.

### Aequorin reconstitution and measurement of [Ca^2+^]_i_

*Arabidopsis thaliana* plants expressing cytosolic apoaequorin were used for [Ca^2+^]_i_ measurements [33,43]. Seedlings were grown on half-strength Murashige and Skoog medium for 7 days. Reconstitution of aequorin was performed *in vivo* by spraying seedlings with 240 µL of 10 µM coelenterazine per Petri dish followed by incubation at 21°C in the dark for 8 hr. Treatments and aequorin luminescence imaging were performed at room temperature using a ChemiPro HT system that includes a cryogenically cooled and back-illuminated CCD camera, liquid nitrogen autofiller, camera controller, and computer-equipped WinView/32 software (Roper Scientific) as described previously [33]. The CCD camera has a 1300 × 1340 pixel resolution and is cooled to −110°C by the cryogenic cooler system prior to image recording. The recording was started 50 s prior treatments and luminescence images were taken every 10 sec. The total remaining aequorin was estimated by treating plants with a discharging solution containing 0.9 M CaCl_2_ in 10% (v/v) ethanol and recorded for 5 min until values were within 1% of the highest discharge value [8,15,33]. WinView/32 and Meta Morph 6 were used to analyze recorded luminescence images. Experiments were carried out at room temperature (22 to 24°C).

### NaCl and H_2_O_2_ treatments

For stress treatments, Petri dishes were placed individually into the ChemiPro HT chamber and luminescence images were taken at 10 sec intervals starting 50 sec prior the treatment. The treatment solution (100 mL) at described concentrations of NaCl or H_2_O_2_ was added into Petri dish in the dark, and luminescence was recorded continuously. For changes in bath solution, a four-channel peristaltic pump (Dynamax RP-1, Rainin) was used to perfuse Petri dish with water as indicated in the figures. Then, additional stress treatment was applied by adding 100 mL solution into Petri dish.

### Calibration of calcium measurements

The cytosolic free cytosolic Ca^2+^ concentrations were calculated based on the calibration equation described previously [41] with modification to the ChemiPro system. The wild-type *Arabidopsis* expressing aequorin were placed individually in each well in 96-well plates containing ½ MS medium, 1.5% (w/v) sucrose, and 0.8% (w/v) agar for 10 days. Kinetic luminescence measurements were performed with an automated microplate luminescence reader (*Mithras* LB 940, Berthold Technologies). After automatic injection of 0.2 ml of solution into the each well bioluminescence counts were integrated every 1 sec as described previously [41]. The solutions containing a range of [NaCl] from 0 to 600 mM were used to treat the plants, and the peak values of [Ca^2+^]_i_ were calculated used the equation described previously [41]. Similar measurements were carried out using the ChemiPro HT system as described above to obtain L/L_max_ values for each treatment, where L is luminescence and L_max_ is the total remaining counts for bioluminescence. Then, we fit these data to the previously describe equation pCa = a * (−log (L/L_max_)) + b, and obtained the equation pCa = 0.9057209 * (−log (L/L_max_)) + 4.7712743. Note that, the calculated Ca^2+^ concentrations presented in the current study are similar to those reported previously [8,41].

## Supporting Information

Figure S1 **H_2_O_2_ levels do not affect [Ca^2+^]_i_ increases in response to NaCl treatment.** (A) *Arabidopsis* seedlings were treated with water (Control), the NADPH oxidase inhibitor DPI (15 µM), and ROS scavenger ascorbic acid (5 mM) and glutathione (5 mM) two hours prior to the NaCl treatment. The seedlings were then subjected to a 200 mM NaCl treatment, and aequorin luminescence was recorded continuously through the treatments in the dark. (B) Quantification of peak [Ca^2+^]_i_ increases from experiments as in (A). Data for three independent experiments are shown (mean ± sd; *n* = 35 to 62; NS, not significant, *P* > 0.05).
(PDF)
