# Supplementary figures and images for: Relationship between NaCl- and H2O2-Induced Cytosolic Ca2+ Increases in Response to Stress in Arabidopsis
Source: PLoS One. 2013 Oct 4;8(10):e76130. doi: 10.1371/journal.pone.0076130 (PMC3790670; doi:10.1371/journal.pone.0076130)

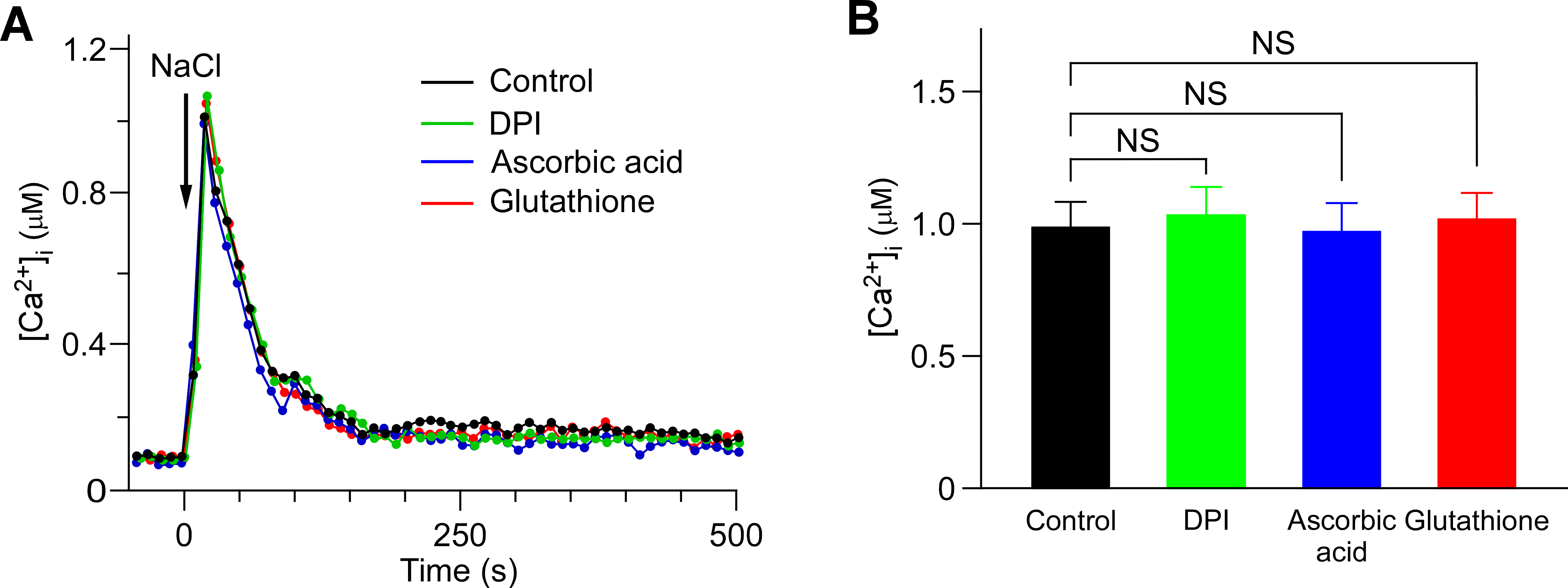

Supplement: Figure S1 — H2O2 levels do not affect [Ca2+]i increases in response to NaCl treatment. (A) Arabidopsis seedlings were treated with water (Control), the NADPH oxidase inhibitor DPI (15 µM), and ROS scavenger ascorbic acid (5 mM) and glutathione (5 mM) two hours prior to the NaCl treatment. The seedlings were then subjected to a 200 mM NaCl treatment, and aequorin luminescence was recorded continuously through the treatments in the dark. (B) Quantification of peak [Ca2+]i increases from experiments as in (A). Data for three independent experiments are shown (mean ± sd; n = 35 to 62; NS, not significant, P > 0.05). (PDF) [file pone.0076130.s001.pdf]
